# Epigenetic Regulation of Macrophage Marker Expression Profiles in Kawasaki Disease

**DOI:** 10.3389/fped.2020.00129

**Published:** 2020-04-03

**Authors:** Mindy Ming-Huey Guo, Ling-Sai Chang, Ying-Hsien Huang, Feng-Sheng Wang, Ho-Chang Kuo

**Affiliations:** ^1^Department of Pediatrics, Kaohsiung Chang Gung Memorial Hospital and Chang Gung University, College of Medicine, Kaohsiung, Taiwan; ^2^Kawasaki Disease Center, Kaohsiung Chang Gung Memorial Hospital and Chang Gung University, College of Medicine, Kaohsiung, Taiwan; ^3^Graduate Institute of Clinical Medical Sciences, College of Medicine, Chang Gung University, Kaohsiung City, Taiwan; ^4^Department of Medical Research, Kaohsiung Chang Gung Memorial Hospital and Chang Gung University, College of Medicine, Kaohsiung, Taiwan; ^5^Core Laboratory for Phenomics & Diagnostics, Kaohsiung Chang Gung Memorial Hospital and Chang Gung University, College of Medicine, Kaohsiung, Taiwan

**Keywords:** Kawasaki disease, macrophage, methylation, transcriptome array, methylation array

## Abstract

Kawasaki disease (KD) is a common systemic vasculitides in children younger than 5 years of age. Activated macrophages are key drivers of vascular inflammation in KD. The aim of this study was to examine differences in M1 and M2 macrophage marker expression in patients with KD. Blood samples were obtained from 18 healthy controls and 18 patients with KD at 24 h prior and 21 days after to intravenous immunoglobulin therapy. GeneChip Human Transcriptome Array 2.0 and Illumina HumanMethylation450 BeadChip were used to examined the mRNA expression and corresponding CpG site methylation ratios of 10 M1 surface markers and 15 M2 surface markers. Of the markers examined 2 M1 markers (TLR2, IL2RA) and 8 M2 markers (ARG1, CCR2, TLR1, TLR8, TLR5, MS4A6A, CD36, and MS4A4A) showed increased mRNA expression in the acute phase of KD which decreased after IVIG therapy (*P* < 0.05). Corresponding CpG sites in the promoter regions these markers were hypomethylated in the acute phase of KD and significantly increased after IVIG therapy. In conclusion, both M1 and M2 markers showed increased mRNA expression in the acute phase of KD. CpG site methylation may be one of the mechanisms governing macrophage polarization in KD.

## Introduction

Kawasaki disease (KD) is one of the most common systemic vasculitides in children younger than 5 years of age. First described by Dr. Tomisaki Kawasaki in 1967 (1), characteristic symptoms include fever for more than 5 days and at least 4 out of five signs of mucocutaneous inflammation including: oral mucosal redness or inflammation, non-suppurative conjunctivitis, lymphadenopathy, swelling of the hands or feet or a polymorphous rash (2). If inadequately treated, patients with KD have a high risk of developing coronary artery aneurysms, sometimes resulting in lifelong anticoagulation and an increased risk of myocardial infarction and other cardiovascular complications later in life (3). Intravenous immunoglobulin therapy (IVIG) in particular, if given within the first 10 days after disease onset, has been found to significantly reduce the risk of coronary artery lesions to <5%. However, up to 10–20% of patients with KD do not response to initial IVIG therapy (2), and have an increased risk of developing coronary artery aneurysms (3). The search for alternative therapies is therefore greatly needed, particularly for patients who are IVIG resistant.

Although the precise immunopathogenesis of KD is unclear, histopathology of the coronary arteries has revealed clues to the immunologic sequence of events that lead to coronary artery dilatation and destruction in patients with KD. Edema of the tunica media layer of the coronary arteries begins around the 6th to 8th day after disease onset. This is then followed by increased infiltration of lymphocytes, monocytes, and neutrophils by the 10th day of disease, which then infiltrates all layers of the coronary artery. This panarteritis is characterized by the formation of proliferative granulomatous inflammation consisting mostly of monocytes and macrophages (4, 5). Macrophages in particular are key drivers of vascular inflammation in KD, producing inflammatory cytokines such as Tumor necrosis factor-α(TNF-α), vascular endothelial growth factor, and proteases such as matrix metalloproteinase-2 (MMP-2) and MMP9 which may destroy elastin fibers and structural components within the arterial wall (6). This in turn results in the development of coronary artery aneurysms beginning at about the 12th day after disease onset. These findings point to the pivotal role aberrantly activated macrophages play in the formation of coronary artery aneurysms in KD.

Macrophages are considered an important component of innate immunity and may initiate inflammation and subsequently adaptive immune responses. Recently it has been found that activated macrophages can also be divided into M1 and M2 subsets, which mirror the Th1 and Th2 polarization seen in T-helper cells (7). Polarization of macrophages into either the M1 or M2 phenotype is highly dependent on the local tissue environment. Classically activated macrophages, also known as M1 macrophages are induced by either IFN-γ alone or in combination with microbial stimuli such as lipopolysaccharides (LPS) or cytokines including tumor necrosis factor (TNF). M1 macrophages produce effector molecules such as reactive oxygen and nitrogen intermediates and also inflammatory cytokines including IL-1β, TNF, and IL-6, thereby amplifying the inflammatory response. They are also capable of inducing Th1 cells and thereby increasing immune responses against intracellular pathogens and increasing tumor resistance (8). In contrast, alternatively activated macrophages, or M2 macrophages, are induced by IL-4, IL-13, IL-10, or immune complexes, produce low levels of inflammatory cytokines, are capable of inducing Th2 cells and are considered immune-modulatory in nature (7).

To date, there have been no studies that specifically examine the role of macrophage M1/M2 polarization in patients with KD. Results from the limited number of existing studies regarding macrophage polarization in other forms of autoimmune vasculitis reveal that both M1 and M2 activation may be involved (8). As an example, in giant cell arteritis, vasculitis of the temporal arteries contain both M1 (inducible nitric oxide positive) and M2 (CD163 positive) macrophages (9, 10). In light of the paucity of research regarding the role of macrophage polarization and the development of KD, the aim of this study was to examine differences in M1 and M2 macrophage marker expression in the patients with acute and resolving KD.

## Materials and Methods

### Subject Recruitment

Initially, a total of 18 patients with KD and 18 healthy controls were enrolled in this study for Human Transcriptome array analysis, and another 12 healthy controls and 12 patients with KD were enrolled for Human Methylation Beadchip analysis. We then enrolled an additional 30 healthy controls and 30 patients with KD for confirmation of initial screening results with RT-PCR, 10 of which were analyzed by pyrosequencing.

Patients were diagnosed with Kawasaki disease if they fulfilled the criteria proposed by the American Heart Association, namely fever which persists for more than 5 days and at least four of the following five clinical criteria: oral mucosal involvement (such as injected or fissured lips, strawberry tongue), bilateral non-exudative conjunctival injection, cervical lymph node enlargement of over 1.5 cm in diameter, changes in the peripheral extremities (edema of the hands and feet, erythema over the plantar, or palmar area in the acute phase or periungual desquamation in the convalescent phase), and a polymorphous rash (2). Patients with Kawasaki disease were then given at least one dose of IVIG (2 g/kg/dose) over a 12 h period after diagnosis in accordance with current clinical guidelines (2). This study has been approved by the Chang Gung Medical Foundation Institutional Review Board (IRB No.:101-0680A3), and conforms to the principles outlined in the Declaration of Helsinki. Informed consent was obtained from the parents or guardians of all patients included in this study.

### DNA and RNA Extraction From Peripheral Blood Samples

Blood samples from the KD group were obtained at two time points: the first blood draw was performed within 24 h prior to the initial dose of IVIG therapy during the acute phase of KD (KD1), and the second blood draw was performed at least 21 days after initial IVIG therapy during the convalescent phase (KD2). All blood samples were centrifuged and the buffy coat containing white blood cells was then removed for further experiments. Total RNA was then extracted from white blood cells according to manufacturer's instructions (mirVana™ miRNA Isolation Kit, Catalog number: AM1560, Life Technologies, Carlsbad, CA). Total RNA samples were checked for quality and quantity and all RNA samples were confirmed to have a RIN (RNA integral number) of ≥7 as previously described (11). Total genomic DNA was extracted according to manufacturer's instructions (Gentra extraction kit). After quantification of all DNA samples was performed by using NanoDrop ND-1000, 500 ng of genomic DNA was taken from each sample for further bisulfite conversion using Zymo EZ DNA Methylation kit (Zymo Research) and stored at 20°C until further use (12).

### Gene Expression Level Profiling With Human Transcriptome Array

For unbiased results, 18 mRNA samples each from the healthy control group (HC group), KD1 group (KD patients 24 h prior to IVIG therapy) and the KD2 group (KD patients 21 days after IVIG therapy) were combined into three pooled RNA libraries each containing six RNA samples. We then used GeneChip® Human Transcriptome Array 2.0 (HTA 2.0, Affymetrix, Santa Clara) to determine gene expression profiles of macrophage surface markers which are commonly expressed on M1 and M2 macrophages after literature review (13–16). Prior to hybridization to the HTA 2.0 microarray chips, all RNA samples were prepared according to manufacturer instructions with the WT PLUS Reagent kit. The hybridized HTA 2.0 microarray chips then underwent quality control inspection. All chips passed quality control examination and were then analyzed using commercial software specific for microarray data analysis (Partek, St. Louis).

### DNA Methylation of M1 and M2 Macrophage Surface Markers

DNA samples taken from the 12 healthy controls, and the 12 Kawasaki disease patients at two time points, 24 h prior to IVIG therapy (KD1) and 21 days after IVIG therapy (KD2) were then used to examine DNA methylation patterns of M1 and M2 macrophage surface markers. Two hundred nanograms of bisulfite-converted genomic DNA were then applied to the Illumina HumanMethylation450 (M450K) BeadChip assay as described in previously (11, 17). Cytosine methylation percentages (β values) were then calculated for each CpG marker within in the promoter region of all M1 and M2 markers included.

### Confirmation of mRNA Expression With Reverse Transcription Polymerase Chain Reaction (RT-PCR)

We then selected the following markers for RT-PCR confirmation of mRNA expression levels: IL1R1, TLR2, TLR4, IL1T2, ARG1, and TLR5. All mRNA samples were first transformed into cDNA according to manufacturer instructions (cDNA-High Capacity cDNA Reverse Transcription kit, Applied Biosystems, Cat. 4368813). Samples were then prepared by adding 2.5 ng/μL of cDNA with 0.2 μL each of forward and reverse primers (10 μM) and 5 μL of SYBR Green PCR Master Mix (ABI, Cat. 4367659) before performing quantitative RT-PCR on the LightCycler R 480 Real-Time PCR System (Roche Molecular Systems, Inc. Indiana, USA). 18S was used as an internal control. IL1R1—Forward (F) AAAGATGACAGCAAGACACCTG, Reverse (R):GTTTGCAATCCTTACCACGCAA; TLR2—(F): GAGTTCTCCCAGTGTTTGGTGT (R):CACACCATCAGAACCCTGTC; IL1R2—(F):ATGACACCCACATAGAGAGCG (R):GAAGAGCGAAACCCACAGAGT; ARG1—(F):ACTTAAAGAACAAGAGTGTGATGTG (R) CGCTTGCTTTTCCCACAGAC; TLR5—(F):TGCTACTGACAACGTGGCTT (R):CCAGGAAAGCTGGGCAACTA; TLR4—(F):ATGCCAGGATGATGTCTGCC (R):TGGATTTCACACCTCCACGC; 18S—(F):GTAACCCGTTGAACCCCATT (R):CCATCCAATCGGTAGTAGCG. We then calculated the relative quantification of mRNA expression levels by using the comparative threshold cycle (CT) method (i.e., by comparing the RT-PCR cycle number required to reach target fluorescence) by using the following equation: 2^−(ΔCTtarget−ΔCTcalibrator)^, also known as 2^−ΔΔCT^.

### Pyrosequecing of Selected CpG Sites

To confirm the percentage of methylation of selected CpG, further pyrosequencing was performed in a separate cohort. First 0.5 μg of genomic DNA underwent bisulfite modification according to manufacturer instructions (EZ DNA methylation kit, Zymo Research) and with eluted in Tris buffer (10 mM) for a final volume of 20 μl. Polymerase chain reaction (PCR) with a reaction mixture containing bisulfite-converted DNA, PyroMark PCR Master Mix (Qiagen), and biotinylated forward and reverse primers of the selected CpG sites. Primers used are as follows: IL1R1 (cg05886087) F:TTTTTGGAAATAGATTGTTTAAGAATGAGA, R:ATAATAATAACTTCCTTCCTTCTAATACAC; TLR2 (cg06618866) F: AGGTTGGGTAGAAGAAGAGAGT, R: AACACTTAACTTTCCCTATAATTACCA; IL1R2 (cg20340242) F: ATTGTAGTTTGGGGGTGT, R: TTCACCTCCAACAAAAACTCA; ARG1 (cg01699630) F: TTAGGGTTGGAAGGGATGTGATA, R:AACCAAAAAATAACAATCAACAATCTTAC; TLR5 (cg05858079) F:AGGGTTTTAGAGTTTTTTTAGGGTAGTA, Reverse:AATAACTTATCCCCAATCACTTTC; TLR4 (cg13730105) F:GAGGGAAAGTTTAGAGGAGTT, R:ACCACCCATTCACAAAACCACTAC. We then calculated the percentage of methylation at for all CpG sites by comparing relative peak height differences at each site (18).

### Statistical Analysis

Statistical analysis was performed using SPSS version 22.0 (SPSS, Inc., Chicago, USA). Data from HTA 2.0 microarray was compared using one-way ANOVA and Bonferroni Correction was used to account for multiple testing. Beta-values from the M450K Array were first transformed into M-values by using the following equation: M-value = log2(beta-value / 1- beta-value) (19). M-values of selected CpG sites were then compared with student's *t*-test for the comparison of healthy controls and patients with KD; and paired *t*-test was used to compare data from KD patients at 2 time points, 24 h prior to IVIG therapy and 21 days after IVIG therapy. Likewise, for data obtained from RT-PCR and pyrosequencing, students *t*-test was used to compare data from healthy controls and KD patients, and paired *t*-test was used to compare data from patients with KD from 2 different time points. Data are presented as mean ± standard error and a *p* < 0.05 was considered as statistically significant. The datasets analyzed during the current study are available from the corresponding author on reasonable request.

## Results

### M1 Marker and M2 mRNA Expression in KD Patients and Controls Using HTA 2.0 Microarray Analysis

**Figure 1:**
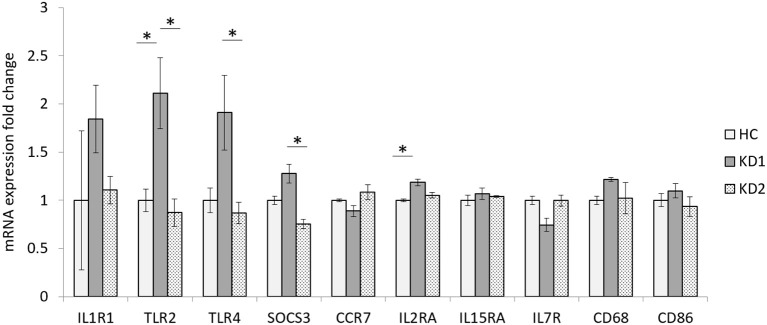
Comparison of M1 macrophage marker mRNA expression fold change by between healthy controls and patients with KD. HC, Healthy controls (18 patients); KD1, acute Kawasaki disease 24 h prior to IVIG therapy (18 patients); KD2, resolving Kawasaki disease 21 days after IVIG therapy (18 patients). An asterisk denotes a *p* < 0.05. Data are expressed as mean ± standard error.

In this study we examined the mRNA expression of 10 M1 surface markers (IL1R1, TLR2, TLR4, SOCS3, CCR7, IL2RA, IL15A, IL7R, CD68, CD86) in three groups, healthy control (HC), acute KD (KD1) and in KD patients 21 days after IVIG therapy (KD2) (Figure 1). We found that of the 10 M1 surface markers examined, 2 markers TLR2, and IL2RA had significantly elevated mRNA expression in the acute phase of KD (mRNA expression fold change HC vs. KD1 1 ± 0.118 vs. 2.113 ± 0.367, *p* = 0.023; 1 ± 0.013 vs. 1.185 ± 0.034, *p* = 0.011, presented as mean ± standard error, respectively). IVIG therapy resulted in a significant decrease in expression of three genes including TLR2, TLR4, and SOCS3 (KD1 vs. KD2 2.113 ± 0.367 vs. 0.873 ± 0.142, *p* = 0.009; 1.911 ± 0.388 vs. 0.868 ± 0.113, *p* = 0.021, 1.276 ± 0.095 vs. 0.755 ± 0.048, *p* =0.032 presented as mean ± standard error, respectively).

**Figure 2:**
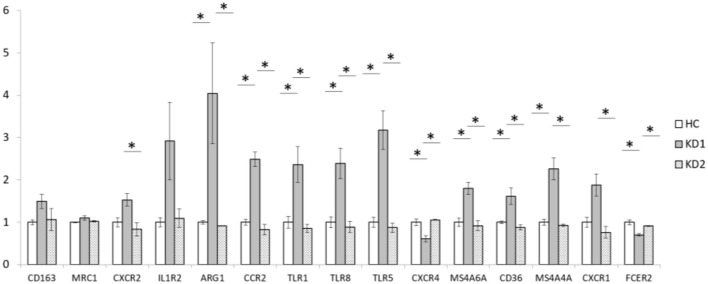
Comparison of M2 macrophage marker mRNA expression fold change by between healthy controls and patients with KD. HC, Healthy controls (18 patients); KD1, acute Kawasaki disease 24 h prior to IVIG therapy (18 patients); KD2, resolving Kawasaki disease 21 days after IVIG therapy (18 patients). An asterisk denotes a *p* < 0.05. Data are expressed as mean ± standard error.

We also examined the mRNA expression of the 15 M2 cell surface markers (CD163, MRC1, CXCR2, IL1R2, ARG1, CCR2, TLR1, TLR5, TLR8, CXCR4, MS4A6A, CD36, MS4A4A, CXCR1, and FCER2) in both KD patients and healthy controls (Figure 2). Of the 15 M2 cell surface markers examined, 8 markers (ARG1, CCR2, TLR1, TLR8, TLR5, MS4A6A, CD36, and MS4A4A) had significantly increased mRNA expression in acute phase KD (HC vs. KD1 1 ± 0.036 vs. 4.046 ± 1.185, *p* < 0.029; 1 ± 0.069 vs. 2.491 ± 0.171, *p* = 0.001; 1 ± 0.134 vs. 2.364 ± 0.423, *p* = 0.013; 1 ± 0.126 vs. 2.390 ± 0.360, *p* = 0.011; 1 ± 0.120 vs. 3.178 ± 0.459, *p* < 0.002; 1 ± 0.099 vs. 1.799 ± 0.143, *p* = 0.011; 1 ± 0.029 vs. 1.616 ± 0.197, *p* = 0.045; 1 ± 0.070 vs. 2.261 ± 0.257, *p* = 0.003 presented as mean ± standard error, respectively), which were then significantly decreased after IVIG therapy (KD1 vs. KD2 4.046 ± 1.185 vs. 0.914 ± 0.003, *p* = 0.001; 2.491 ± 0.172 vs. 0.828 ± 0.121, *p* < 0.001; 2.363 ± 0.423 vs. 0.854 ± 0.094, *p* = 0.005; 2.390 ± 0.360 vs. 0.885 ± 0.131, *p* = 0.005; 3.178 ± 0.459 vs. 0.872 ± 0.106, *p* < 0.001; 1.799 ± 0.143 vs. 0.916 ± 0.117, *p* = 0.005; 1.616 ± 0.197 vs. 0.879 ± 0.057, *p* = 0.013; 2.261 ± 0.257 vs. 0.929 ± 0.027, *p* = 0.002; presented as mean ± standard error, respectively). One marker, CXCR2 showed a statistically significant decrease in mRNA expression after IVIG therapy (KD1 vs. KD2 1.528 ± 0.151 vs. 0.834 ± 0.158, *p* = 0.038). Two markers, CXCR4 and FCER2, had decreased mRNA expression in the acute phase of KD (HC vs. KD1 1 ± 0.078 vs. 0.609 ± 0.071, *p* = 0.006; 1 ± 0.059 vs. 0.703 ± 0.033, *p* = 0.004 presented as mean ± standard error, respectively) which increased after IVIG therapy (KD1 vs. KD2 0.609 ± 0.071 vs. 1.057 ± 0.011, *p* = 0.003; 0.703 ± 0.033 vs. 0.912 ± 0.012, *p* = 0.022 presented as mean ± standard error, respectively).

### Confirmation of mRNA Expression Levels Using RT-PCR

**Figure 3:**
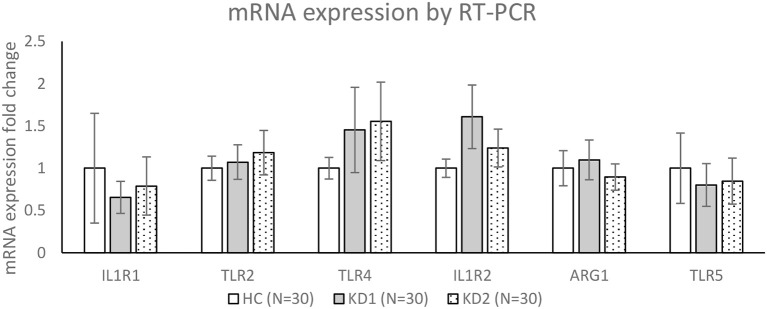
Confirmation of mRNA expression levels by RT-PCR. HC, Healthy controls (30 patients); KD1, acute Kawasaki disease 24 h prior to IVIG therapy (30 patients); KD2, resolving Kawasaki disease 21 days after IVIG therapy (30 patients). Data are expressed as mean ± standard error.

We then chose three M1 markers (IL1R1, TLR2, TLR4) and three M2 markers (IL1R1, ARG1, TLR5) with the highest fold change between acute KD (KD1) and healthy controls (HC), for RT-PCR confirmation of mRNA expression levels in a separate cohort of 30 KD patients and 30 healthy controls (Figure 3). For the three M1 markers selected, TLR2 and TLR4 mRNA expression levels were higher on average in the acute KD group (HC vs. KD1 1 ± 0.143 vs. 1.073 ± 0.204, *p* = 0.771, 1 ± 0.128 vs. 1.452 ± 0.504 *p* = 0.388 presented as mean ± standard error, respectively), which increased after IVIG therapy (KD1 vs. KD2 1.073 ± 0.204 vs. 1.184 ± 0.262, *p* = 0.737, 1.452 ± 0.504 vs. 1.554 ± 0464, *p* = 0.878 presented as mean ± standard error, respectively), although these changes were not statistically significant. IL1R1 on the other hand, was lower in the acute phase of KD (KD1) (HC vs. KD1 1 ± 0.649 vs. 0.655 ± 0.190, *p* = 0.612 presented as mean ± standard error), and increased after IVIG therapy (KD1 vs. KD2 0.655 ± 0.190 vs. 0.789 ± 0.344 *p* = 0.739 presented as mean ± standard error), but these changes were again not statistically significant.

For the three M2 markers selected, IL1R2 and ARG1 showed higher average expression in acute KD (KD1) (HC vs. KD1 1 ± 0.108 vs. 1.607 ± 0.375, *p* = 0.129, 1 ± 0.207 vs. 1.098 ± 0.235, *p* = 0.754 presented as mean ± standard error, respectively), which decreased after IVIG therapy (KD1 vs. KD2 1.607 ± 0.375 vs. 1.238 ± 0.223, *p* = 0.334, 1.098 ± 0.235 vs. 0.895 ± 0.155, *p* = 0.319 presented as mean ± standard error, respectively), although these changes were not statistically significant. TLR5 showed lower expression in the acute phase of KD (HC vs. KD1 1 ± 0.416 vs. 1.098 ± 0.252, *p* = 0.685, presented as mean ± standard error), which increased slightly after IVIG therapy (KD1 vs. KD2 1.098 ± 0.252 vs. 0.847 ± 0.272, *p* = 0.881, presented as mean ± standard error), but was also not statistically significant.

### Methylation Levels of Corresponding M1 and M2 Marker CpG Sites

We then used Infinium HumanMethylation 450 BeadChip (Illumina) to examine the CpG site methylation trends of the 10 M1 and 15 M2 surface markers included in this study (full results in Tables 1, 2). Because hypomethylation of CpG sites in the promoter region of genes leads to increased gene mRNA expression (20), we tried to identify corresponding CpG sites which were hypomethylated in genes with increased mRNA expression.

**Table 1:** Comparison of CpG site methylation fold changes of M1 macrophage markers in healthy controls and patients with Kawasaki Disease.

**Gene symbol**	**Target ID**	**Fold-Change (KD1 vs. HC)**	***p*-value (KD1 vs. HC)**	**Fold-Change (KD2 vs. HC)**	***p*-value (KD2 vs. HC)**	**Fold-Change (KD2 vs. KD1)**	***p*-value (KD2 vs. KD1)**
IL1R1	cg05886087	−1.21732	0.000006*	−1.05198	0.041622*	1.15716	0.000431*
	cg06880612	−1.10287	0.000006*	−1.04512	0.000028*	1.05526	0.001306*
	cg06943668	−1.00203	0.981921	−1.01157	0.016226*	−1.00952	0.020868*
	cg09363443	−1.04316	0.000003*	−1.02102	0.009032*	1.02168	0.026471*
	cg19754707	−1.01321	0.005930*	−1.02287	0.000020*	−1.00954	0.019204*
TLR2	cg02345613	−1.00936	0.001359*	−1.01118	0.081380	−1.0018	0.775385
	cg03523945	−1.01976	0.000002*	−1.00686	0.058977	1.01281	0.021652*
	cg03610073	−1.0096	0.142018	−1.03862	0.003742*	−1.02875	0.009883*
	cg06405222	−1.00235	0.978750	−1.02161	0.000003*	−1.01922	6.44E-05*
	cg06618866	−1.0313	0.000083*	−1.02138	0.013046*	1.00971	0.3621
	cg15852258	1.00144	0.701207	1.01416	0.000083*	1.0127	0.001127*
	cg16547110	1.0028	0.484426	−1.00836	0.026797*	−1.01118	0.000831*
	cg17916835	−1.01637	0.012428*	−1.01335	0.051072	1.00299	0.381223
	cg19037167	−1.02192	0.000062*	−1.01232	0.020365*	1.00948	0.021885*
TLR4	cg04061482	1.00672	0.766085	−1.0013	0.958727	−1.00803	0.031436*
	cg05429895	−1.01334	3.51E-07*	1.0092	0.008932*	1.02266	2.55E-06*
	cg13730105	−1.15383	0.000035*	−1.0344	0.125541	1.11546	0.000666*
SOCS3	cg01897823	1.00161	0.174351	1.0103	0.000612*	1.00868	0.005838*
	cg03752138	−1.04133	0.000013*	−1.08298	1.61E-10*	−1.04	9.92E-06*
	cg04548563	−1.00384	0.157371	1.00241	0.243839	1.00626	0.000491*
	cg04610187	−1.07261	0.000012*	−1.09513	3.61E-07*	−1.02099	0.011633*
	cg10368834	−1.01261	0.500669	−1.01755	0.313913	−1.00488	0.159929
	cg12999453	1.00176	0.756747	1.00261	0.327255	1.00085	0.491749
	cg14253096	−1.00833	0.263096	−1.00123	0.900064	1.00708	0.434305
	cg14496305	−1.02192	0.028009*	−1.0291	0.000009*	−1.00703	0.000825*
	cg14721618	1.00797	0.028757*	−1.03565	4.00E-11*	−1.0439	1.03E-07*
	cg15502888	1.00195	0.173635	1.01376	0.000056*	1.01178	0.000944*
	cg16253629	−1.00525	0.363686	−1.00862	0.133850	−1.00335	0.619161
	cg18538958	−1.01177	0.116957	−1.04835	0.000455*	−1.03616	0.004421*
	cg18855780	1.00358	0.054970	−1.00029	0.496837	−1.00387	0.011058*
	cg21500342	−1.01037	0.001759*	−1.00208	0.502114	1.00827	0.023568*
	cg22749855	1.04158	0.000056*	−1.03244	0.003358*	−1.07537	5.71E-06*
	cg23985214	−1.00508	0.111600	1.00529	0.070625	1.0104	0.000407*
	cg26747885	1.01678	0.001820*	−1.01665	0.000177*	−1.0337	9E-06*
CCR7	cg07248223	1.19679	0.000020*	1.01481	0.484177	−1.17932	0.000231*
	cg08724510	−1.00393	0.420426	−1.01055	0.246296	−1.00659	0.678295
	cg13270626	−1.00996	0.010916*	−1.01008	0.086185	−1.00011	0.578922
	cg13504059	1.04995	0.005605*	−1.00974	0.577696	−1.06018	0.001253*
	cg16047279	1.22107	0.000005*	1.03122	0.180002	−1.18411	0.000176*
	cg17067993	−1.00135	0.830882	1.01779	0.000009*	1.01916	6.75E-05*
	cg26960939	1.12657	0.000234*	1.00808	0.677247	−1.11754	0.000322*
IL2RA	cg10486505	−1.00264	0.267540	1.00034	0.969791	1.00298	0.404473
	cg11733245	1.01058	0.003400*	1.02302	0.000089*	1.01231	0.0232*
	cg16949914	1.04095	0.001556*	1.01301	0.333223	−1.02758	0.028752*
	cg26105232	−1.22018	0.000002*	−1.05966	0.042674*	1.15148	0.000197*
	cg26316423	1.03938	0.008769*	−1.00981	0.516062	−1.04957	0.000267*
	cg27131821	1.00412	0.159932	1.01061	0.000098*	1.00645	0.031687*
IL15RA	cg00508950	−1.00036	0.989579	−1.00867	0.392564	−1.00831	0.282137
	cg01718738	−1.01164	0.066812	−1.05996	7.07E-09*	−1.04776	2.66E-06*
	cg03691156	−1.00144	0.280415	−1.00355	0.000505*	−1.0021	0.009006*
	cg05144147	−1.00041	0.777642	−1.01095	0.000867*	−1.01054	0.002767*
	cg08676905	−1.0012	0.508908	1.0071	0.017166*	1.00831	0.002231*
	cg09290866	−1.04138	0.000096*	−1.02537	0.028776*	1.01561	0.18332
	cg10686550	−1.01486	0.000056*	−1.00163	0.580957	1.01321	0.007886*
	cg13730379	−1.02743	0.000268*	−1.10803	5.56E-12*	−1.07845	1.67E-07*
	cg15071696	−1.00745	0.000153*	−1.01708	0.000065*	−1.00956	0.077432
	cg19979643	−1.00974	0.001077*	1.0096	0.001914*	1.01944	4.11E-05*
	cg24348573	−1.09086	0.000021*	−1.03349	0.046193*	1.05552	0.006376*
	cg24677732	−1.00015	0.690520	−1.00477	0.001312*	−1.00463	0.001144*
IL7R	cg01804183	1.10724	0.000005*	−1.00733	0.648630	−1.11535	1.11E-05*
	cg04312209	1.1607	0.000017*	1.06724	0.013245*	−1.08758	0.000639*
	cg27582180	−1.0079	0.027828*	−1.01512	0.002740*	−1.00717	0.128131
CD68	cg01934632	−1.00606	0.018303*	−1.00528	0.030173*	1.00077	0.780858
	cg02346901	−1.02463	0.000072*	−1.03887	0.000001*	−1.0139	0.112221
	cg02613380	−1.00316	0.322148	−1.00648	0.157876	−1.0033	0.380751
	cg04510815	−1.0012	0.230227	−1.05302	0.000001*	−1.05175	0.000165*
	cg05209483	−1.17957	0.000002*	−1.06637	0.008743*	1.10615	0.000196*
	cg05270696	−1.00707	0.234327	−1.054	1.15E-09*	−1.0466	9.25E-07*
	cg05652328	−1.02215	0.010914*	−1.02657	0.011688*	−1.00432	0.647877
	cg06852395	−1.03391	0.006797*	1.01227	0.219162	1.0466	6.35E-05*
	cg07421515	−1.02056	0.008227*	1.01715	0.023434*	1.03806	5.47E-05*
	cg10587621	−1.03019	0.000017*	1.0162	0.115484	1.04688	0.000478*
	cg13749033	−1.0266	0.000005*	−1.04487	4.77E-08*	−1.01779	0.008606*
	cg15095917	−1.17028	0.000003*	−1.06479	0.005198*	1.09907	0.00052*
	cg16056611	1.00386	0.119835	1.00356	0.522221	−1.0003	0.677193
	cg16547235	−1.00794	0.001642*	−1.02074	0.000032*	−1.0127	0.040127*
	cg18900669	−1.11258	0.000012*	−1.0362	0.040849*	1.07371	0.001219*
	cg21808406	−1.15626	0.000027*	−1.03643	0.163403	1.11562	0.000321*
	cg22259724	1.00184	0.699657	−1.05907	1.18E-08*	−1.06102	2.5E-06*
	cg24219033	−1.00705	0.127193	−1.10006	1.93E-09*	−1.09236	6.89E-06*
	cg27010959	−1.00357	0.073877	1.00212	0.524067	1.0057	0.080075
CD86	cg01021483	1.00741	0.925748	−1.00276	0.457571	−1.01018	0.040495*
	cg01436254	−1.02563	0.031212*	1.00976	0.445134	1.03563	0.000256*
	cg01878435	−1.01772	0.003025*	−1.00637	0.236206	1.01127	0.024484*
	cg04387658	−1.0469	0.000221*	−1.05514	0.000120*	−1.00788	0.332995
	cg04880737	−1.03367	0.000270*	−1.0287	0.140235	1.00483	0.639097
	cg06327732	−1.01266	0.035835*	−1.09175	3.86E-08*	−1.07809	9.93E-05*
	cg07108581	−1.00402	0.571690	−1.01501	0.036759*	−1.01095	0.053199
	cg11874272	1.00599	0.580171	−1.02365	0.023441*	−1.02978	0.00443*
	cg13069531	−1.09587	0.000010*	−1.02491	0.118781	1.06923	0.000181*
	cg13617155	−1.07336	3.95E-08*	−1.01786	0.081353	1.05452	5.63E-05*
	cg16331599	−1.03419	0.000019*	1.03103	0.017603*	1.06628	0.000159*

*HC, Healthy controls (12 patients), KD1, acute Kawasaki disease 24 h prior to IVIG therapy (12 patients), KD2, resolving Kawasaki disease 21 days after IVIG therapy (12 patients). An asterisk denotes a p < 0.05. Data are expressed as mean ± standard error*.

**Table 2:** Comparison of CpG site methylation fold changes of M2 macrophage markers in healthy controls and patients with Kawasaki Disease.

**Gene symbol**	**Target ID**	**Fold–Change (KD1 vs. HC)**	***p*–value (KD1 vs. HC)**	**Fold–Change (KD2 vs. HC)**	***p*–value (KD2 vs. HC)**	**Fold–Change (KD2 vs. KD1)**	***p*–value (KD2 vs. KD1)**
CD163	cg05939324	−1.00564	0.607439	−1.03034	8.19E-09*	−1.02457	0.000004*
	cg07264679	−1.02182	0.001080*	−1.00376	0.442244	1.018	0.004770*
CXCR2	cg00002473	−1.0051	0.169105	−1.12953	3.17E-12*	−1.1238	5.84E-08*
	cg03464560	−1.01415	0.042228*	1.01414	0.035118*	1.02849	0.000457*
	cg06547715	−1.21994	0.000003*	−1.08806	0.004337*	1.1212	0.001580*
	cg10591797	−1.04278	0.000003*	−1.02701	0.002627*	1.01535	0.013146*
	cg13739417	−1.02656	1.51E-07*	−1.00984	0.038776*	1.01656	0.000003*
	cg14150666	−1.09239	0.000017*	−1.05641	0.004698*	1.03405	0.056927
	cg17081998	−1.02885	0.000021*	−1.01866	0.000020*	1.01	0.049850*
	cg19225688	−1.15693	0.000002*	−1.1111	0.000010*	1.04125	0.081946
	cg25941354	−1.14501	0.000001*	−1.0989	0.000003*	1.04196	0.026826*
IL1R2	cg01716667	1.0002	0.774800	1.00303	0.302792	1.00283	0.439499
	cg01820934	−1.05541	0.000496*	−1.04066	0.000002*	1.01417	0.656054
	cg08879111	1.03957	0.021655*	−1.00696	0.617709	−1.04681	0.016664*
	cg12910851	1.05778	0.000246*	−1.0293	0.082031	−1.08877	0.000187*
	cg14325025	−1.1719	0.000002*	−1.05687	0.008913*	1.10884	0.000907*
	cg15400471	−1.00467	0.364754	−1.04807	1.24E-09*	−1.0432	0.000009*
	cg17142183	−1.1585	0.000002*	−1.06719	0.004062*	1.08556	0.007689*
	cg20340242	−1.1908	0.000011*	−1.06805	0.004795*	1.11493	0.002271*
	cg20470722	1.00181	0.531958	1.00174	0.426552	−1.00007	0.968432
	cg20856504	−1.01286	0.022020*	−1.02197	0.000400*	−1.00899	0.047466*
	cg21603144	−1.16457	0.000003*	−1.06695	0.004560*	1.09149	0.002574*
	cg25920665	−1.00515	0.163200	−1.08262	8.02E-14*	−1.07707	2.07E-08*
ARG1	cg01699630	−1.27067	0.000006*	−1.07096	0.013553*	1.18648	0.000784*
	cg02862362	−1.0219	0.000423*	−1.05761	5.22E-09*	−1.03494	0.000836*
	cg04639555	−1.01709	0.048098*	−1.09702	4.20E-08*	−1.07859	0.000009*
	cg06975018	−1.21034	0.000006*	−1.10044	0.001201*	1.09987	0.012810*
	cg16178743	−1.02082	0.000970*	−1.04491	0.000001*	−1.0236	0.001477*
CCR2	cg03928384	1.03628	0.000361*	−1.00716	0.535037	−1.0437	0.025890*
	cg04110105	−1.02183	0.013246*	1.05256	0.000014*	1.07554	0.000001*
	cg06585307	−1.01249	0.001057*	−1.00794	0.115795	1.00451	0.318164
	cg07824265	1.00619	0.845400	1.00272	0.597202	−1.00346	0.660749
TLR1	cg02016764	1.01476	0.192395	1.03396	0.146065	1.01892	0.552275
	cg08757862	−1.01801	0.000255*	−1.01152	0.017786	1.00642	0.146685
	cg09316306	−1.05901	4.44E-08*	1.00289	0.840879	1.06207	0.000025*
	cg22839308	−1.01643	0.000005*	1.00323	0.336331	1.01972	0.000031*
TLR8	cg00741717	−1.03908	0.004612*	−1.04154	0.001406*	−1.00236	0.868754
	cg07759587	−1.18112	0.000183*	−1.07489	0.005605*	1.09883	0.007118*
	cg13153942	−1.04969	0.000121*	−1.17152	2.16E-08*	−1.11606	0.000323*
TLR5	cg00255925	1.00094	0.724266	−1.02269	0.000009*	−1.02364	0.000001*
	cg01181681	1.05831	0.050795	−1.02191	0.226850	−1.0815	0.002321*
	cg03702975	1.0162	0.025828*	−1.00228	0.701607	−1.01852	0.014849*
	cg04219417	1.04379	0.007797*	−1.02969	0.072505	−1.07478	0.000045*
	cg05696109	−1.00085	0.899629	1.0164	0.000280*	1.01726	0.000233*
	cg05858079	−1.01157	0.025045*	−1.03379	0.000113*	−1.02197	0.009294*
	cg07015886	1.05173	0.018117*	−1.04237	0.007657*	−1.09629	0.000078*
	cg07538512	−1.00577	0.008527*	1.00509	0.282785	1.01089	0.004198*
	cg07574686	−1.00294	0.547926	−1.00489	0.589048	−1.00194	0.844872
	cg09025215	1.04164	0.002594*	−1.00674	0.685264	−1.04867	0.001783*
	cg12275981	1.02507	0.001353*	1.00879	0.257961	−1.01615	0.083901
	cg12900151	−1.01145	0.561033	−1.07805	0.004476*	−1.06584	0.000014*
	cg13557530	−1.00724	0.036950*	−1.06435	7.88E-09*	−1.0567	0.000035*
	cg14015211	−1.00305	0.288593	−1.02824	1.15E-07*	−1.02511	4.64E-07*
	cg14228103	1.02911	0.649290	−1.04962	0.242205	−1.08018	0.000703*
	cg17599809	1.00068	0.204633	1.00472	0.012943*	1.00403	0.003094*
CXCR4	cg01150411	1.00506	0.249898	−1.03548	6.45E-09*	−1.04072	0.000001*
	cg01679081	−1.01258	0.003439*	−1.00938	0.131749	1.00317	0.879754
	cg02367708	−1.00685	0.120859	1.05452	0.000005*	1.06175	2.83E-08*
	cg02902079	1.00481	0.005133*	1.01141	0.000008*	1.00658	0.002594*
	cg04513185	1.00501	0.602533	−1.01922	0.001923*	−1.02433	0.002504*
	cg05303524	−1.00251	0.240073	1.01704	0.000026*	1.01959	0.000039*
	cg06679534	−1.00207	0.009328*	1.00728	0.000080*	1.00937	0.000001*
	cg07784959	1.03481	0.001492*	1.00939	0.405797	−1.02519	0.034825*
	cg10020290	−1.01579	0.103705	−1.03207	0.122123	−1.01602	0.904041
	cg10718991	−1.00136	0.204748	1.00433	0.264240	1.0057	0.059655
	cg12311057	−1.00072	0.923893	1.02168	0.000089*	1.02241	0.000095*
	cg12595667	−1.094	0.000188*	1.015	0.213481	1.1104	0.000055*
	cg13075444	1.00835	0.890689	1.01556	0.211114	1.00715	0.179608
	cg14341558	−1.00002	0.891365	−1.00541	0.008769*	−1.00539	0.001941*
	cg15629460	−1.01642	0.002006*	−1.00823	0.223973	1.00812	0.006788*
	cg16675708	−1.00436	0.075055	1.00814	0.012377*	1.01253	0.003664*
	cg17398233	−1.00147	0.261895	1.02067	2.47E-07*	1.02216	0.000010*
	cg18443412	−1.01099	0.023076*	1.00747	0.312259	1.01854	0.009974*
	cg19238531	1.00042	0.509786	1.0132	0.000001*	1.01278	0.000010*
	cg20366284	1.01161	0.002974*	1.02284	0.001200*	1.0111	0.078279
	cg20823742	−1.00807	0.025481*	−1.00004	0.998787	1.00802	0.062154
	cg20824211	−1.00134	0.908299	1.00446	0.114488	1.00581	0.036624*
	cg22376465	−1.00422	0.299725	1.05887	1.43E-08*	1.06334	3.19E-07*
	cg22923827	−1.00025	0.853380	1.02835	0.000002*	1.02861	0.000013*
	cg23374992	−1.12467	0.000058*	−1.03645	0.024458*	1.08512	0.002011*
	cg23885472	1.00005	0.609237	−1.04216	1.55E-12*	−1.04222	3.97E-09*
	cg26191576	−1.02438	0.004549*	−1.02045	0.016970*	1.00385	0.230726
	cg26425669	−1.00038	0.970585	1.01703	0.000070*	1.01742	0.000935*
	cg26548542	−1.00298	0.640367	−1.00034	0.947957	1.00263	0.706639
MS4A6A	cg00673646	−1.028	0.000001*	−1.01275	0.016401*	1.01506	0.020049*
	cg03055440	−1.0639	0.000177*	−1.04838	0.000674*	1.01481	0.107192
	cg04353769	−1.22143	0.000003*	−1.09082	0.003478*	1.11973	0.001009*
	cg05893353	−1.03021	0.000357*	−1.0289	0.000546*	1.00128	0.552353
	cg05995607	−1.13031	2.05E-07*	−1.06293	0.001182*	1.06339	0.000064*
	cg06881914	−1.18836	0.000007*	−1.07923	0.002601*	1.10111	0.002903*
	cg09552517	−1.00065	0.835430	−1.00237	0.102849	−1.00172	0.344976
	cg20284999	−1.00459	0.138725	−1.02688	0.000441*	−1.02219	0.010529*
	cg26250585	−1.23688	0.000002*	−1.08552	0.001559*	1.13943	0.000222*
MS4A4A	cg01229998	−1.03957	0.001109*	−1.03609	0.000101*	1.00336	0.459733
	cg02893799	−1.02222	0.000912*	1.00069	0.920470	1.02292	0.000049*
	cg03841312	−1.02138	0.094573	1.00158	0.758436	1.02299	0.087823
	cg08564601	−1.11027	2.86E-07*	−1.05303	0.000514*	1.05435	0.001913*
	cg12604169	−1.00689	0.179323	−1.09988	1.91E-11*	−1.09235	2.35E-07*
	cg13350775	−1.00083	0.753622	−1.03844	0.000004*	−1.03758	0.000091*
	cg16952963	−1.01145	0.499234	1.02103	0.284685	1.03272	0.019107*
	cg18025430	−1.09764	0.000003*	1.00365	0.809193	1.10164	0.000019*
	cg23754934	−1.06136	0.003444*	−1.06635	0.000279*	−1.0047	0.711363
	cg27008678	1.00064	0.759894	−1.09093	0.000001*	−1.09163	0.000002*
CD36	cg05917188	1.02088	0.000404*	−1.07051	3.81E-07*	−1.09287	1.11E-07*
	cg10207609	−1.03718	0.000611*	−1.02136	0.109615	1.01549	0.101425
	cg14093018	−1.00448	0.332432	−1.03382	0.000835*	−1.02921	0.009032*
	cg14479884	−1.06917	0.000012*	−1.05796	0.000423*	1.0106	0.091037
	cg15383705	1.0018	0.585782	−1.01006	0.059004	−1.01188	0.014507*
	cg18433146	−1.00851	0.103165	−1.0955	9.88E-09*	−1.08625	0.000001*
	cg18508525	−1.01533	0.000764*	−1.00922	0.058274	1.00605	0.089320
	cg19096849	−1.0014	0.731414	−1.04094	3.84E-07*	−1.03948	0.000063*
	cg21055948	−1.00666	0.076670	−1.05859	1.56E-10*	−1.05159	3.65E-07*
	cg25783969	−1.00568	0.140078	−1.03079	0.000008*	−1.02497	0.000296*
	cg27625491	1.01064	0.044850*	−1.04251	0.000001*	−1.0536	0.000072*
CXCR1	cg00832199	−1.04132	0.000014*	1.00947	0.122855	1.05118	0.000056*
	cg01218945	−1.00254	0.085168	−1.00439	0.058763	−1.00184	0.467406
	cg06683602	−1.08885	0.000663*	−1.0358	0.128723	1.05122	0.011153*
	cg07016356	−1.01573	0.033727*	1.01907	0.019301*	1.0351	0.000039*
	cg09294937	1.00932	0.044589*	−1.00548	0.452138	−1.01485	0.028151*
	cg13048967	−1.10816	0.000013*	−1.01322	0.247233	1.0937	0.000082*
	cg13519373	−1.04173	0.000103*	1.00969	0.113034	1.05182	0.000027*
	cg14702787	1.00427	0.259337	−1.04983	3.98E-08*	−1.05432	0.000008*
	cg15426604	1.00342	0.680212	−1.00075	0.986117	−1.00418	0.733233
	cg15768138	−1.23706	0.000001*	−1.06298	0.019749*	1.16376	0.000040*
	cg15908708	−1.1178	0.000002*	−1.00354	0.722836	1.11385	0.000035*
	cg18467756	−1.04023	0.000322*	−1.07416	0.000001*	−1.03262	0.000725*
	cg21004129	−1.08522	0.000055*	−1.02151	0.139107	1.06237	0.001292*
FCER2	cg02796568	1.00445	0.295506	−1.00339	0.423123	−1.00785	0.032055*
	cg03221619	1.0228	0.032891*	−1.02625	0.016817*	−1.04964	0.000054*
	cg05489904	1.00099	0.850242	−1.02234	0.023137*	−1.02335	0.011843*
	cg05641903	−1.16318	0.000025*	−1.04813	0.038301*	1.10977	0.002059*
	cg09773499	−1.04717	0.000005*	−1.01668	0.070251	1.02999	0.000100*
	cg10488777	−1.00702	0.015982*	−1.00947	0.039725*	−1.00243	0.705201
	cg12261095	−1.07231	0.000001*	−1.07899	2.80E-08*	−1.00623	0.778167
	cg12387247	−1.04274	0.000491*	−1.0523	0.000635*	−1.00917	0.535602
	cg20234640	−1.00111	0.778083	−1.03487	0.007825*	−1.03372	0.010365*
	cg26040211	1.0026	0.260376	1.00999	0.061587*	1.00737	0.287632

*HC, Healthy controls (12 patients), KD1, acute Kawasaki disease 24 h prior to IVIG therapy (12 patients), KD2, resolving Kawasaki disease 21 days after IVIG therapy (12 patients). An asterisk denotes a p < 0.05. Data are expressed as mean ± standard error*.

Of the three M1 markers with the highest fold change in mRNA expression on HTA 2.0, we identified three corresponding CpG sites the highest negative beta-value fold change between KD1 and HC groups, indicating that these sites were likely to be hypomethylated. Selected CpG sites included cg05886087 (KD1 vs. HC beta-value fold change −1.217, *p* < 0.001) within the IL1R1 gene, cg06618866 (KD1 vs. HC beta-value fold change −1.0313, *p* < 0.001) within the TLR2 gene, cg 13730105 (KD1 vs. HC beta-value fold change −1.154, *p* < 0.001) within the TLR4 gene. Likewise, for the M2 markers three corresponding CpG sites if the highest negative beta-value fold change were selected including cg20340242 (KD1 vs. HC beta-value fold change −1.191, *p* < 0.001) within the IL1R2 gene, cg01699630 (KD1 vs. HC beta-value fold change −0.1271, *p* < 0.001) within the ARG1 gene, cg05858079 (KD1 vs. HC beta-value fold change −1.011, *p* = 0.025).

**Figure 4:**
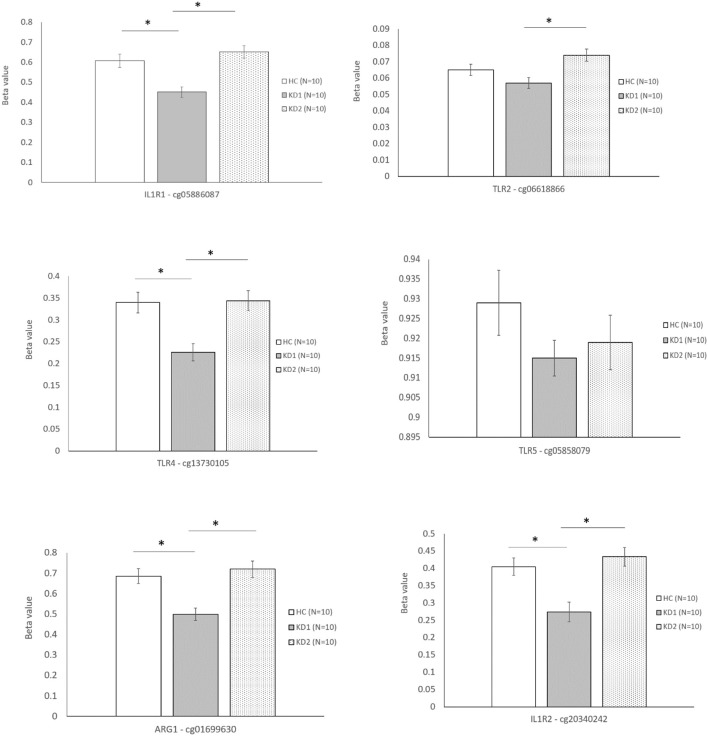
Beta values of selected markers after pyrosequencing. HC, Healthy controls (10 patients); KD1, acute Kawasaki disease 24 h prior to IVIG therapy (10 patients); KD2, resolving Kawasaki disease 21 days after IVIG therapy (10 patients). An asterisk denotes a *p* < 0.05. Data are expressed as mean ± standard error.

The six selected CpG sites were then confirmed with a separate cohort of 10 healthy controls, and 10 patients with KD by pyrosequencing. Four of the CpG sites selected corresponding to the M1 markers IL1R1 (cg05886087), TLR4 (cg13730105), and the M2 markers IL1R2 (cg20340242) and ARG1 (cg01699630) were significantly hypomethylated in the acute KD group (HC vs. KD1, 0.607 ± 0.0322 vs. 0.451 ± 0.026, *p* = 0.001; 0.340 ± 0.023 vs. 0.226 ± 0.019, *p* = 0.002; 0.405 ± 0.025 vs. 0.274 ± 0.028, *p* = 0.003; 0.686 ± 0.036 vs. 0.499 ± 0.030, *p* = 0.001; beta values presented as mean ± standard error.), and became hypermethylated after IVIG therapy (KD1 vs. KD2 0.451 ± 0.026 vs. 0.652 ± 0.031, *p* < 0.001; 0.226 ± 0.019 vs. 0.344 ± 0.022, *p* < 0.001; 0.274 ± 0.028 vs. 0.434 ± 0.027, *p* < 0.001; 0.499 ± 0.030 vs. 0.720 ± 0.039, *p* < 0.001; beta values presented as mean ± standard error.) The CpG site selected for the M1 marker TLR2 (cg06618866) showed significant hypermethylation after IVIG therapy (KD1 vs. KD2 0.057 ± 0.033 vs. 0.074 ± 0.004, *p* < 0.001). There were no significant changes in methylation of the Cpg site selected for the M2 marker TLR5, cg05858079 (Figure 4). These results suggest that changes in gene methylation may be one of the many mechanisms governing M1 and M2 polarization in patients with KD.

## Discussion

In the first portion of our study we utilized HTA 2.0 microarray analysis to identify M1 and M2 markers with the highest degree of mRNA expression fold-change. Of the 10 M1 markers examined in our study, only two had significantly higher mRNA expression in the acute phase of KD which decreased after IVIG therapy. In contrast, a higher percentage of M2 surface markers, eight out of the 15 surveyed showed significantly increased mRNA expression in the acute phase of KD which decreased after IVIG therapy. Markers with the highest degree of mRNA fold change include the M1 markers TLR2, TLR4, and IL1R1, and the M2 markers TLR5, IL1R2, and ARG1. Expression levels of the six markers with the highest mRNA expression fold changes were then confirmed in a separate cohort of 30 patients with KD and 30 healthy controls by RT-PCR. Of the six selected markers, only IL1R2 and ARG1, both M2 markers were found to have elevated mRNA expression levels in the acute phase of KD which decreased after IVIG therapy, although these results were not statistically significant.

Because CpG site hypomethylation is one of the many mechanisms which regulate gene expression, we then examined the methylation data of corresponding M1 and M2 marker gene CpG sites included on M450K array. Six CpG sites with the highest degree of hypomethylation fold-change corresponding to the three M1 (IL1R1, TLR2, TLR4) and three M2 markers (IL1R2, TLR5, ARG1) with the highest mRNA expression fold change on HTA 2.0 were selected for confirmation. Pyrosequencing of the six CpG sites selected was performed in a separate cohort of 10 KD patients and 10 healthy controls. Four of the CpG sites selected corresponding to the M1 markers IL1R1 (cg05886087), TLR4 (cg13730105), and the M2 markers IL1R2 (cg20340242) and ARG1 (cg01699630) were significantly hypomethylated in the acute KD group and were then hypermethylated after IVIG therapy, which mirrored the increased mRNA expression of these genes on HTA 2.0 microarray. This suggests that CpG site methylation may be one of the many mechanisms which regulate M1 and M2 macrophage polarization in KD.

In our previous research, we have found that both Th-1 and Th2 subset activation are involved in the acute phase of KD. Th1 associated cytokines including IL-2 IL-6, TNF-α, and IFN-γ were significantly increased in the acute phase of KD and decrease after IVIG therapy (21–23). In addition, lower levels of TNF-α prior to IVIG therapy was associated with a lower risk of IVIG resistance, and higher levels of IL-6 levels after IVIG therapy was associated with a higher risk of coronary artery lesions (21). Similarly, Th2 associated cytokines including IL-4, IL-5, and IL-10 are elevated in the acute phase of KD (21, 23, 24), although patients with higher IL-5 and eosinophil counts after IVIG therapy were less likely to develop coronary artery lesions (24). The results of these studies suggest that while both Th1 and Th2 activity are associated with the acute phase of KD, higher levels of Th1 activity may increase the risk of coronary artery lesions, while increased Th2 activity may protect against them. Because M1 and M2 macrophage activation increases Th1 and Th2 immunity respectively, we initially hypothesized that both M1 and M2 macrophage activity would increase in the acute phase of KD.

Monocyte derived macrophages can be to activated into either M1 or M2 macrophages according to the tissue microenvironment. As mentioned earlier, stimuli such as IFN-γ, TNF-α or LPS promotes macrophage polarization toward the M1 phenotype, and IL-13 and IL-4 promotes macrophage polarization into the M2 phenotype. In addition, there appears to be plasticity among the M1/M2 phenotypes; once a macrophage is activated into the M1 phenotype, it can still be repolarized into the M2 phenotype according to dynamic changes in the tissue microenvironment, and vice versa (25). In this study we also found that although both M1 and M2 markers are elevated in the acute phase of KD, a higher percentage of M2 markers were elevated.

Both M1 and M2 marker mRNA expression decreased after IVIG therapy. Although there are no other studies to date that specifically examine M1 and M2 macrophage activation in KD, one study of 28 patients with KD showed that CD14+ CD16+ monocytes were elevated in the acute phase of KD (26). CD14+ and CD16+ monocytes are considered to be “intermediate-type” monocytes which can differentiate into both M1 and M2 macrophages, and are highly associated with IL-10 production (27). Likewise, both M1 and M2 macrophages appear to be implicated in the development of other autoimmune vasculitis. As an example, in a murine model of systemic lupus erythematosus (SLE), M1 macrophages are recruited to the kidney early after kidney injury and create a pro-inflammatory environment that aids with the clearance of damaged or apoptotic cells (28). In addition, M2b macrophages, an immune-regulatory M2 macrophage subtype, can be induced by incompletely phagocytosed immune complexes in lupus patients, and are associated with the development of lupus in both mouse models and in patients with SLE glomerulonephritis (29). Although the most direct way of examining macrophage polarization would be to perform immunohistochemical staining of the coronary arteries, specimens can only be obtained from patients who have died of KD. Therefore, we can only hypothesize that both M1 and M2 macrophages are activated in the acute phase of KD. It is possible that M2 macrophages, due to their role in tissue healing and repair are responsible for the resolution of KD, although further functional studies are required.

Of the 10 M1 macrophage markers and 15 M2 macrophage markers examined in this study, we found that the M1 markers TLR2, TLR4, and IL1R1 and the M2 markers TLR5, IL1R2, and ARG1 had the highest levels of mRNA expression in the acute phase of KD. Previous research has found that toll-like receptors, particularly toll-like receptor 2 (TLR2) is associated with the development of KD. In human patients with KD, increased expression of TLR2 positive CD14 monocytes occurs in the acute phase of KD (22), and is down regulated after IVIG therapy (30), and can successfully predict risk of coronary artery lesions and IVIG resistance (31). In mouse models of KD, mice which were TLR2 or MyD88 deficient failed to develop KD after induction by Lactobacillus casei cell-wall extract (LCCWE), suggesting that TLR2 signaling through the MyD88 pathway is crucial for the development of KD (32). In addition to TLR2, we also found that TLR4 and TLR5 had high levels of mRNA expression in the acute phase of KD. TLR2, TLR4, and TLR5 are capable of recognizing pathogen associated molecular patterns (PAMPs) including lipoteichoic acid, lipopolysaccharides, and flaggellin on bacteria and aids the phagocytosis of pathogens and further signal transduction and amplification of the immune response through the MyD88 pathway (33). These results lend credence to the hypothesis that bacterial infections may pose a possible trigger for the development of KD (34).

Both IL1R1 and IL1R2 belong to the interleukin-1 receptor family. IL1R1 which is expressed on M1 macrophages, acts as a receptor of the cytokines IL-1a, IL-1b, and IL-1 receptor antagonist (IL-1RA). Conversely, IL1R2 which is expressed on M2 macrophages, acts as a decoy receptor for IL-1a, IL-1b, and IL-1RA thereby inhibiting the further signal transduction of these cytokines (35). The IL-1 pathway is a crucial part of the development of KD, as evidenced by one study which examined the whole blood transcriptional profiles of 146 subjects with KD, and found that all of the top five pathways upregulated in KD included IL-1 signaling molecules such as IL1R1 and IL1R2 (36). IL-1α and IL-1β signaling is also essential to the development of a mouse model of KD (37), and treatment with IL-R antagonist (Anakinra) has been shown to inhibit arterial aneurysm formations in mouse models (38).

Furthermore, we found that the M2 marker ARG1 showed significant increase of mRNA expression in the acute phase of KD. ARG1 diverts arginine away from the inflammatory iNOS (inducible nitric oxide synthase) pathway, thereby modulating inflammatory responses (39). Although there have been no studies which specifically examine ARG1 expression in KD, there have been many studies that show a link between enhanced iNOS expression in the acute phase of KD and the development of coronary artery disease (40, 41) perhaps suggesting that increased ARG1 expression in acute KD may be associated with protection against coronary artery lesions.

Finally, we found that four CpG sites corresponding to the M1 markers IL1R1 (cg05886087), TLR4 (cg13730105), and the M2 markers IL1R2 (cg20340242) and ARG1 (cg01699630) were significantly hypomethylated in the acute KD group and were then hypermethylated after IVIG therapy. Previous studies have found that epigenetic regulation of gene expression particularly through DNA methyltransferases (DNMT) may be one of the ways which regulate M1 and M2 macrophage polarization. DNMTs are enzymes that bind methyl groups to DNA strands, and include DNMT1, which maintains DNA methylation by adding methyl groups to hemimethylated strands of DNA during replication; and DNMT3a and DNMT3b, which add methyl groups to previously unmethylated DNA (i.e., *de novo* methylation). There is evidence that increased expression of DNMT1 promotes polarization toward the M1 macrophage phenotype possibly through regulation of the PPAR-γ pathway (42). In a mouse model of obesity, dietary supplementations of saturated fatty acids significantly increased DNMT1 expression and macrophages and increased methylation of the promoter region of PPAR-γ. This led to a decrease in PPAR-γ expression, a transcriptional factor that promotes macrophage polarization toward the alternatively activated M2 phenotype, therefore suppressing M2 macrophage polarization. These findings were corroborated in another study, where macrophage specific overexpression of DNMT1 in a transgenic mouse model of atherosclerosis led to decreased PPAR-γ expression and increased inflammatory macrophage activation and increased atherosclerosis. The researchers also found that DNMT1 expression was negatively correlated to PPAR-γ expression in the peripheral monocytes of human subjects with atherosclerosis (43, 44). Conversely, M2 macrophages have higher levels of expression of DNMT3a and DNMT3a1 which may be associated with AMP-activated protein kinase (AMPK) signaling (42, 45).

Because DNA and RNA samples were extracted from whole white blood cells (including neutrophils, lymphocytes and monocytes), the heterogeneity of cells used is one of the limitations of our study. In our study we found that acute Kawasaki disease (KD1) was associated with a higher percentage of neutrophils (KD1 vs. HC 55.28 ± 2.79% vs. 31.05 ± 1.95%), and a lower percentage of lymphocytes (KD1 vs. HC 33.62 ± 2.56% vs. 59.75 ± 2.14%) when compared to the healthy controls. The ratios of neutrophils and lymphocytes normalized and were comparable to healthy controls 21 days after IVIG therapy (KD2), and the percentage of monocytes was similar in all three groups (HC 5.6 ± 0.50%, KD1 5.8 ± 0.46%, KD2 4.8 ± 0.37%). In our study we found that a much higher percentage of M2 related markers showed increased mRNA expression in the acute phase of KD, but decreased after IVIG therapy, particularly IL1R2, TLR5, and ARG1. While TLR5 is also expressed on neutrophils, it is more highly expressed in monocytes (46). Likewise, while IL1R2 is also expressed on B cells and T cells, it is more densely expressed on monocytes (47, 48).

In conclusion, this is the first study to specifically examine the role of M1 and M2 macrophage polarization in patients with KD. We found that both M1 and M2 markers showed increased expression in the acute phase of KD which decreased after IVIG therapy, with the M1 markers TLR2, TLR4, and IL1R1 and the M2 markers TLR5, IL1R2, and ARG1 showing the highest levels of mRNA expression. We also found that the corresponding promoter CpG sites of these markers were hypomethylated in the acute phase of KD, and hypermethylated after IVIG therapy, which suggest that CpG site methylation may be one of the mechanisms governing macrophage polarization in KD. Further functional studies are needed to confirm the role of macrophage polarization in the development of KD, which may prove to be a novel therapeutic target in the future.

## Data Availability Statement

The data analyzed in this study can be found in https://www.ncbi.nlm.nih.gov/geo/query/acc.cgi?acc=GSE109430 and https://www.ncbi.nlm.nih.gov/geo/query/acc.cgi?acc=GSE109351.

## Ethics Statement

The studies involving human participants were reviewed and approved by Chang Gung Medical Foundation Institutional Review Board. Written informed consent to participate in this study was provided by the participants' legal guardian/next of kin.

## Author Contributions

MG participated in the study design, analyzed data, and wrote the main manuscript. L-SC, Y-HH, and F-SW participated in data collection and analysis. H-CK was responsible for study designs and edited the main manuscript. All authors have reviewed the manuscript prior to publication.

### Conflict of Interest

The authors declare that the research was conducted in the absence of any commercial or financial relationships that could be construed as a potential conflict of interest.
